# Changes in time-use and drug use by young adults in poor neighbourhoods of Greater Buenos Aires, Argentina, after the political transitions of 2001-2002: Results of a survey

**DOI:** 10.1186/1477-7517-8-2

**Published:** 2011-01-20

**Authors:** Diana Rossi, Dhan Zunino Singh, María Pía Pawlowicz, Graciela Touzé, Melissa Bolyard, Pedro Mateu-Gelabert, Milagros Sandoval, Samuel R Friedman

**Affiliations:** 1 Intercambios Civil Association. Av. Corrientes 2548 Piso 2 Dto. D - C1046AAP - Ciudad de Buenos Aires - Argentina; 2 National Development and Research Institutes, Inc. (NDRI). 71 West 23rd Street, 8th Floor, 10010 New York, NY - USA; 3 Emory University. 400 Ashbury Drive, 30306 Atlanta, GA - USA

## Abstract

**Background:**

In some countries, "Big Events" like crises and transitions have been followed by large increases in drug use, drug injection and HIV/AIDS. Argentina experienced an economic crisis and political transition in 2001/2002 that affected how people use their time. This paper studies how time use changes between years 2001 and 2004, subsequent to these events, were associated with drug consumption in poor neighbourhoods of Greater Buenos Aires.

**Methods:**

In 2003-2004, 68 current injecting drug users (IDUs) and 235 young non-IDUs, aged 21-35, who lived in impoverished drug-impacted neighbourhoods in Greater Buenos Aires, were asked about time use then and in 2001. Data on weekly hours spent working or looking for work, doing housework/childcare, consuming drugs, being with friends, and hanging out in the neighbourhood, were studied in relation to time spent using drugs. Field observations and focus groups were also conducted.

**Results:**

After 2001, among both IDUs and non-IDUs, mean weekly time spent working declined significantly (especially among IDUs); time spent looking for work increased, and time spent with friends and hanging out in the neighbourhood decreased.

We found no increase in injecting or non-injecting drug consumption after 2001. Subjects most affected by the way the crises led to decreased work time and/or to increased time looking for work--and by the associated increase in time spent in one's neighbourhood--were most likely to increase their time using drugs.

**Conclusions:**

Time use methods are useful to study changes in drug use and their relationships to every day life activities. In these previously-drug-impacted neighbourhoods, the Argentinean crisis did not lead to an increase in drug use, which somewhat contradicts our initial expectations. Nevertheless, those for whom the crises led to decreased work time, increased time looking for work, and increased time spent in indoor or outdoor neighbourhood environments, were likely to spend more time using drugs. These data suggest that young adults in traditionally less-impoverished neighbourhoods may be more vulnerable to Big Events than those in previously drug-impacted impoverished neighbourhoods. Since Big Events will continue to occur, research on the pathways that determine their sequelae is needed.

## Background

Socio-economic political transitions in Russia, other former Soviet Union states, and Indonesia were followed by severe economic disruption, alienation of youth, growth of injection and non-injection drug use, sexual risk behaviours, female sex work, and a number of epidemics including human immunodeficiency virus (HIV), hepatitis C, and sexually transmitted infections [1-7]. Transitions in the Philippines did not lead to such outcomes. The term "Big Events" [5-8] is a concept that might help us think about wars, transitions and similar phenomena and their impact on drug use and HIV.

The Argentinean Big Events involved large-scale increases in unemployment and poverty, leading to factory seizures, highway blockages by the unemployed and their allies, and eventually widespread massive demonstrations in which middle class, working class, the unemployed, the poor and students drove four successive presidents from office in less than two months.

During the 1980s and 1990s, Argentina struggled with high inflation and industrial stagnation that peaked in the 1990s [9]. By the end of 2001, a large number of Argentina's working class had become impoverished. Argentina's National Centre of Statistics and Census [10] reported by May 2002 poverty had increased to 18 million people, half of the country's total population. The official unemployment rate reached 25%. Job loss and inflation were on the rise and made it increasingly difficult for working class families to meet even their most basic needs. Popular unrest led to mass road blockades and protests by the unemployed, seizures of factories and other workplaces by workers, and to the ousting of four presidents from December 2001 to early 2002.

Time Use: One pathway through which Big Events might lead to drug use and other problems is by changing how young adults use their time. "Time use" measurement can provide data on changes in the lives of young men and women.

North American investigations of adolescents have studied relationships among use of time in daily activities and the risks of alcohol abuse, delinquency, teenage pregnancy, the onset of drug use, and school dropout rates [11-14]. These studies find that existing behavioural risks in youth can be partly attributed to such activities as *hanging out*, especially without monitoring by parents or by other adults. Nevertheless, intense participation in some activities can also lead to risky behaviour, particularly when the activities do not help the personal development of the adolescents or the activities are not of their interest [14]. Researchers have also found a positive correlation between the number of hours worked during the school year and less investment and performance in school, greater psychological and somatic distress, increased drug and alcohol use, and increased delinquency [15].These North American studies have generally been conducted on populations other than those of young adults in poor neighbourhoods. In Latin America, there have been general studies carried out using time use measures in Argentina, Brazil Nicaragua and Mexico [16-21]. Most of these studies collected time use data on "unpaid care work--the work, or 'production', that usually falls mainly on women's shoulders and that includes housework; care at the household level for children, the elderly, sick people and those with disabilities; and voluntary community-oriented work" [22].

Insofar as we are aware, no previous studies have investigated the impact of "big events", such as those in Argentina's recent past on time use in poor drug-impacted neighbourhoods; nor have studies investigated the impact of these events on changes in drug use.

We here describe time use changes between 2001 and 2004 among injecting drug users (IDUs), other drug users, and non-users. We use the data to explore whether, following "Big Events" that took place:

1. The number of IDUs increased.

2. Drug use increased among those whose time working decreased, or whose time spent looking for work decreased.

## Methods

### Study Purpose

These data were collected as part of a study organized rapidly in 2002 to monitor and try to understand the impact of the Argentine Big Events on drug use and HIV risk in Buenos Aires. We were worried that these events might precipitate an HIV outbreak similar to that in Russia and other former Soviet Union countries, and wanted to monitor what was happening in neighbourhoods that seemed to be ripe for such developments. Our overall project goal was to avert a potential disaster if it began to appear; and our secondary purpose was to learn how to prevent HIV outbreaks after future Big Events anywhere in the world. As in many studies organized under emergency conditions, funding was very limited, and this restricted the depth of data collection that could be achieved and the number of subjects who could be recruited.

### Sample and Data Collection

The sample consisted of young adults 21-35 years of age (in Argentina, the legal age of adulthood began at 21 at the time the study was developed). Two categories of participants were enrolled: (1) current injecting drug users, and (2) other youth regardless of their drug use. Approximately equal numbers of men and women were recruited from the local population which is overwhelmingly Hispanic. Recruitment of IDUs was done by outreach workers using the snowball sampling technique to identify hidden populations. The outreach workers were familiar with local IDUs from prior projects.

The study was conducted among young injecting drug users and other young adults in four impoverished drug-impacted neighbourhoods of Avellaneda (Villa Corina, Villa Luján, Dock Sud, Sarandí) in Southern Greater Buenos Aires. Avellaneda was a highly industrial part of the city between 1930 and 1970 [23], but strong de-industrialization between 1975 and 1990 destabilized the region. By the end of the 1990s, Avellaneda had deteriorated into an area with recycled or abandoned infrastructures and industrial equipment. It was no longer a homogeneous, industrial neighbourhood, but was instead marked by social inequality and plagued with very poor living conditions among most of the population [24].

Questionnaires and sampling procedures were developed for two categories of participants: 1. current IDUs who had injected drugs in the last 12 months, and 2. non-IDUs, including non-drug users. Survey data collection began in December 2003 and ended in January 2005. We surveyed 235 non-IDUs and 68 current IDUs. Work occurred between 10 am and 6 pm to assure better security conditions for the working team.

Before recruiting subjects we conducted ethnographic studies of the neighbourhoods to identify places where IDUs live and hang out. Outreach workers who were familiar with local IDUs from prior projects used snowball sampling to identify, recruit, and interview IDUs. We recruited non-injector young adults by four methods: (1) quota-samples from door-to-door recruitment near locations where IDUs lived or hung out (n= 82); (2) street-intercept methods using randomised times to avoid biases based on time of day (n= 66); and (3) respondent-assisted network recruitment of subjects' friends (n= 59); (4) As we neared the end of the survey period, 28 additional local non-IDUs whom the interviewers had come to know were recruited to achieve a better age and gender balance.

Ethnographic methods included nine key informant interviews of IDUs and non-IDUs, elder members of the community, and workers from the community health care centre; two focus groups of male non-IDUs, and two focus groups of male IDUs and former IDUs. Field observations also took place from 2003-2004. This ethnography was conducted by Dhan Zunino Singh, a sociologist who spent several days a week in the field over a two year period. Injection drug use was ascertained with the help of harm reduction staff who had worked in the field since 1999 and knew the drug injectors because of their participation in the program activities.

Participants in all phases of this research signed informed consent forms. All procedures were approved and monitored by the institutional review boards of Fundación Huésped (Buenos Aires) and National Development and Research Institutes (New York). Confidentiality was maintained through assigning code numbers to all interviewees and other materials containing information on subjects. Participants were reimbursed for their time and effort with a voucher they could exchange for food at local supermarkets (approximately US$3).

### Questionnaire and Measures of Drug Use

Questionnaires asked participants about their drug use, sexual practices, networks, norms and time use. Data on time use and drug use at specific times, including before and after the political-economic crisis of 2001, are the focus of this paper.

Major questionnaire items asked included the following variables: sociodemographic and situational variables that included age, gender, homelessness, sexual orientation, marital status, education, living arrangements, other indicators of economic status and of access to and use of health services and experiences with the criminal justice system.

Social influence variables included social support, contact with institutions like church and school, community involvement, and outward influences on others (e.g. telling others to engage in protective behavior such as condom use, or to avoid risky behaviors such as injection drug use or sex with an IDU).

Peer norms towards drug use, drug dealing, sexual behaviors, sex with IDUs and, for IDUs, sex with non-IDUs were measured with items that capture different aspects of what is meant by "norms," since they ask about both (a) actual experiences in which close friends encouraged the respondent to engage in a behavior; and (b) perceived norms (whether they think their close friends would object if they did engage in the behavior).

Sexual risk behaviors: Age of first intercourse. Protected and unprotected sex, number of partners of each sex, drug-use of partners, and sharing drugs with partners were measured for "ever" and for last 3 months.

Drug risk behaviors: Questions were asked about a number of substances they used and their routes of administration. These included the age of first use, and data on frequency of use "ever" and for last 3 months, for: cannabis, intranasal cocaine, non-prescription medicines, alcohol and solvents, as well as different injected drugs. Injecting drugs included cocaine, alcohol, morphine, Ketamine, amphetamines, and any other that the respondent mentioned.

Deliberate AIDS risk reduction in sexual and drug use behaviors.

Self-reported medical history (e.g. prior HIV tests, history of STDs and STD symptoms, drug use treatments).

Sexual and drug injection networks

Participants were interviewed with a 120 items questionnaire with close-ended questions, face-to-face using paper and pencil questionnaires (CASI and Audio-CASI technologies couldn't be used with the time and resources available). It took between 30 to 45 minutes to complete the questionnaire.

### Time Use Measures

In our study we used *stylised time use measures*, also referred to as *stylised questions *or *direct *questions [25-28]. Stylised questions are suitable for measuring time spent on specific activities [25]. Stylised measures ask respondents to provide "normal" or "typical" amounts of time per day, week, month, or year devoted to a particular activity. Respondents are essentially asked to aggregate details of their time into categories of activities [27].

Stylised measures are replicable, so a respondent can be asked about both recent time use and past time use, as has been investigated and validated by the Bureau of Labour Statistics in relation to their Current Population Survey [27]. The reliability of retrospective data collection among drug users can be increased by linking the time periods in question to memorable events [29] (Gerry V. Stimson, personal communication, February 3, 2009). We performed such linkage in relation to the events of the Argentine economic and political crisis.

To effectively conduct a time measures study, it is necessary to have prior knowledge of the daily activities of the population [27]. Our ethnographic research provided such knowledge and helped us define the time measure questions.

For a range of activities, we asked *How many hours do you generally spend per week ...*

• at work,

• looking for work,

• hanging out in the neighbourhood,

• performing childcare/doing house work,

• being with friends,

• using drugs

We asked these questions about the number of hours used for each activity in a typical week at present, and then asked how many hours were used per week for the same activity "three years ago" (which ranged from December 2000 to January 2002). Hence, we obtained the number of hours spent at each activity in (approximately) 2001 and 2004; and this allowed us to observe changes in different facets of everyday life. To measure change in time use, we re-coded each activity to indicate whether the respondent increased, decreased or didn't change the number of hours they used for each activity.

We excluded or analysed separately subjects who responded that, in both 2001 and 2004, they spent "zero hours" in these activities: changes in time of drug use; work; looking for work; and hanging out in the neighbourhood.

In the case of the variable *change in time using drugs*, we excluded those cases reporting no drug use in either period. The variable *change in time working *was collapsed into two categories: (a) Decreased or never worked, (b) No change or increase. For *change in time looking for work*, we analysed those who did not look for work separately.

To measure the time spent in the neighbourhood in 2004, we designed a close-ended question with a range of mutually exclusive answers: *How much time do you spend in the neighbourhood? Would you say you... (Choose one)*

• sleep here, but that's about it?

• sleep here, and spend some free time here, but are gone a lot?

• spend most of your time here, but spend some time elsewhere?

• rarely/never leave the neighbourhood?

We recoded this variable into 3 categories: spent *very little time *(answers 1 and 2), *considerable time *(3) and *most of the time *(4) in the neighbourhood.

### Statistics

Student's t- test was used to analyse differences of means. Kendall's Tau C was used for cross-tabulations with ordinal variables.

## Results

### Socio-demographic aspects

Of the 303 subjects, 55.4% were male and 44.6% were female; 86.8% of IDUs were men (see Table 1). Ages ranged from 21 to 35; the mean age was 27.2. No subjects were homeless; 63.7% had a partner, and almost half had children. Only 21.8% had graduated from high school and 1.3% obtained university degrees. Only 28% of IDUs and 37% of non-IDUs had jobs (p = .107, Fisher).

**Table 1:** Socio-demographic data by IDUs and Non-IDUs

		IDUs	Non-IDUs	total
	***N*** ***%***	***68*** ***100%***	***235*** ***100%***	***303*** ***100%***

**Sex**	Men	59 86.8%	109 46.4%	168 55.4%
	
	Women	9 13.2%	126 53.6%	135 44.6%

**Age**	21-25	13 19.1%	102 43.4%	115 38%
	
	26-30	23 33.8%	74 31.5%	97 32%
	
	31-35	32 47.1%	59 25.1%	91 30%

**Have Work**	No	49 72.1%	148 63%	197 65.0%
	
	Yes	19 27.9%	87 37%	106 35.0%

**Have Partner**	No	33 48.5%	77 32.8%	110 36.3%
	
	Yes	35 51.5%	158 67.2%	193 63.7%

**Education**	None	4 5.9%	20 8.5%	24 7.9%
	
	Primary	49 72.1%	160 68.1%	209 69%
	
	Secondary	15 22.1%	51 21.7%	66 21.8%
	
	University	0 0%	4 1.7%	4 1.3%

**Have children**	No	34 50%	128 54.5%	162 53.5%
	
	Yes	34 50%	107 45.5%	141 46.5%

The poverty line according to Argentina's National Centre of Statistics and Census for 2004 was 700 pesos per month (1 U.S. dollar = 3 pesos); 14% of subjects earned 100 pesos or less, 50% earned between 100 and 300 pesos, and 19% earned between 300 and 500 pesos. (Some respondents were not the only wage earners of the household, however). Incomes were similar between IDUs and non-IDUs, and between men and women.

The main sources of income were temporary jobs (53.3%) and governmental social plans for unemployed heads of household (28%) (see Table 2). Many (27.5%) were supported by their parents; and only 18% had fixed or stable work as a source of income. Very few responded that their sources of income came from illegal activities. Sex work was mentioned in only one case.

**Table 2:** Sources of income by IDUs and Non-IDUs (Multiple responses N = 399)

	IDUs		Non-IDUs		Total	
	
Frequency of Sources of Income	Count of response (N = 89)	Pct of Cases (N = 68)	Count of response (N = 310)	Pct of Cases (N = 234)	Count of responses (N = 399)	Pct of Cases (N = 302)
Temporary jobs	46	67.6%	115	49%	161	53.3%

Social plans for unemployed heads of the household	17	25%	67	28.6%	84	28%

Fixed or stable work	7	10.3%	48	20.5%	55	18%

Supported by their parents	10	14.7%	73	31%	83	27.5%

Illegal activities: theft, drug sales, sex work	9	13%	7	3%	16	5.5%

**Note**: The frequency and percentages that indicate the sources of income were obtained from a table of multiple responses

### Drug Use and Time Use

In the last 12 months, women (77%) were more likely to report they used no drugs than men (34%). The most commonly non-injected drugs used by men were cannabis, cocaine and non-prescription medicines; and by women cannabis and cocaine (see Table 3). Men (32%) were more likely to inject drugs than women (7%).

**Table 3:** Drug use in the last 12 months by Sex

Drug use	Male (N = 168)	Female (N = 135)	Total (N = 303)
None-used	58 34%	104 77%	162 54%

Cannabis	109 65%	30 22%	139 46%

Non-injecting cocaine	85 51%	19 14%	104 34%

Non-prescription medicines	57 34%	9 7%	66 22%

IDU	54 32%	9 7%	63 21%

Solvents	10 6%	1 0.7%	11 4%

Note: Numbers in each row for each drug are the numbers of subjects who gave a given response, remembering that a subject could give many responses. The N's at the top are the numbers of subjects. Percents are the percents of subjects who said they used the drug in the last 12 months.

In Argentina, cocaine is the most commonly injected substance. (Heroin use is very rare). Almost all (98.5%) of IDUs surveyed injected cocaine. Some had also injected amphetamines (33%), alcohol (20%), Ketamine (16%) or morphine (14%) at least once in their lives. Injection of wine and/or other alcoholic beverages has been reported among injection drug users in Argentina and in other Latin American countries [30-33].

Comparing changes in drug use between 2001 and 2004, the number of IDUs and drug users stayed stable or decreased:

1 started sniffing cocaine

1 started injecting drugs

4 people stopped sniffing cocaine (for at least the last 12 months)

6 IDUs stopped injecting (no injection during the last 12 months)

Table 4 (bottom lines) shows the *mean hours *spent using drugs in 2001 and in 2004. Changes differed for non-IDUs and IDUs. The time spent using drugs remained constant at *7 to *8 hours per week for non-IDUs, but mean time spent using drugs declined among IDUs. In 2001, they spent 47 hours per week; in 2004 they spent "only" 35 hours (p = 0.005).

**Table 4:** Change in reported time use *(mean hours) *between 2001* and 2004* by IDUs and Non-IDUs

	IDUs			Non-IDUS		
**Mean hours per week spent...**	2001*	2004*	**Student t**	2001*	2004*	**Student t**

At work	26.09	14.50	*.002*	20.87	15.14	*.001*

Looking for work	7.19	13.28	*.010*	6.56	8.26	*.061*

Being with friends	44.40	34.64	*.030*	26.47	22.97	*.007*

Hanging out in the neighborhood	34.78	25.56	*.027*	18.37	14.70	*.011*

Housework/childcare	16.51	17.94	*.602*	20.5	24.45	*.003*

Using drugs	46.85	34.93	*.005*	7.97	7.24	*.458*

*More precisely, "2004" means at the time of interview, which could also be in December 2003 or January 2005; and "2001" means three years before the interview.

### Changes in Time Use in Other Daily Activities between 2001-2004

Table 4 also shows differences in *mean hours *spent for other activities between 2001 and 2004. Among both IDUs and non-IDUs, *mean weekly time spent at work declined significantly *(for IDUs, from 26 to 14 hours', and for non-IDUs, from 21 to 15 hours) while time spent *looking for work *increased significantly for IDUs (from 7 to 13 hours) and perhaps slightly for non-IDUs (6.6 to 8.3 hours; p= .061). Time spent *being with friends *decreased to 35 hours for IDUs and to 23 hours for non-IDUs; and time *hanging out in the neighbourhood *decreased to 26 hours for IDUs and to 15 hours for non-IDUs.

In our ethnographic work, we observed many young people (generally non-injecting drug users) living or congregating in groups on street corners, in building entrances, and in abandoned public spaces. Cocaine is frequently used at home, but is sometimes publicly consumed; and alcohol, cannabis and inhalants (glue or solvents) often are consumed in public. Currently, non-prescribed medications are rarely used publicly; and public injection drug use generally does not occur. Drug sales take place in private.

Time spent in "housework/childcare" increased among non-IDUs (*p = .003*), though not for IDUs (*p = .602*). Housework and childcare increased significantly for non-IDU men (8.3 hours per week in 2001, 13.7 hours in 2004, *p = .012) *but not for women (31.0 hours peer week in 2001 and 33.7 hours in 2004, p = .109), though clearly remaining much higher among women.

### Changes in Time Use and Drug Use among Non-IDUs

Women use drugs less than men (see Table 3), but when we analysed the *change in time using drugs *among those non-IDUs who reported some drug use, we found no significant differences between men and women (p = .864) (see Table 5).

**Table 5:** Change in time using drugs by Sex among Non-IDUs.

Change in time using drugs		Sex		
		
		Male	Female	Total
Kendall Tau C p. = .864	Decrease	30 54.5%	18 54.5%	48 54.5%
	
	No change	6 10.9%	5 15.2%	11 12.5%
	
	Increase	19 34.5%	10 30.3%	29 33%

	***N*** ***Total***	***55*** ***100%***	***33*** ***100%***	***88*** ***100%***

For non-IDUs, the *mean hours *of drug use remained stable. However, among those non-IDUs who used drugs both in 2001 and 2004, 54% decreased and 33% increased the hours they spent using drugs. Work-related variables seem to have influenced who did which: 43% of those who either did not work or who decreased their work time increased the time they spent using drugs, whereas only 4% of those who either maintained their work time at the same level, or increased the time they worked, spent more time using drugs (see Table 6).

**Table 6:** Changes in reported time using drugs, time working, and time looking for work among non-IDUs

	Change in time working			Change in time looking for work				
	
Change in time using drugs	Decrease or not work	No change or Increase	Total	Not looking for work in either year	Decrease	No Change	Increase	Total
Decrease	28 43.1%	20 87.0%	48 54.5%	18 48.6%	14 82.4%	8 88.9%	8 32%	48 54.5%

No change	9 13.8%	2 8.7%	11 12.5%	5 13.5%	0 0%	1 11.1%	5 20%	11 12.5%

Increase	28 43.1%	1 4.3%	29 33.0%	14 37.8%	3 17.6%	0 0%	12 48%	29 33.0%

***N*** ***Total***	***65*** ***100%***	***23*** ***100%***	***88*** ***100.0%***	***37*** ***100%***	***17*** ***100%***	***9*** ***100%***	***25*** ***100%***	***88*** ***100%***

Kendall Tau C-	p = .000			p = .000				

Note: Subjects included were 88 non-IDUs who were active drug users at the time of interview (which took place between December 2003 and January 2005) and/or three years before the interview.

Similarly, among non-IDUs, time spent using drugs increased among more of those who spent more time looking for work after the crisis (48%) than among those who spent less time looking for work (18%). Time using drugs also increased among those who did *not look for work *in either year (38%) (see Table 6).

## Discussion

### Time use measures

Pearson (1987) and Dorn & South (1987) discussed how in the United Kingdom and the United States a combination of widespread unemployment in a local geographic area with drug distribution networks would lead to widespread heroin use [28,29]. Pearson described this in terms of unemployment disrupting culturally-determined time routines; and he saw heroin use as providing an alternative way to structure one's time that would provide a lifestyle with difficult tasks that would provide a new way to achieve a level of status as a successful drug user. This description parallels what we observe in localities in Avellaneda in which cocaine-use has been fairly common; and ties in with our argument that the Argentine crises might lead to increased drug use and drug injection.

Time use methodology permits both detection and exploration of behavioural changes in societal (macro) and individual (micro) environments [34-36]. It is a method well-suited to studying issues like those that Pearson and Dorn & South raise.

In this study, the use of *stylised measures of time use *helped us to describe changes in the use of drugs between 2001 and 2004, and to analyse those changes in the context of everyday life activities. We recommend the application of time use methods in further research about drug users. In order to facilitate this, we recommend that research be conducted to assess the most reliable and valid ways to ask time use questions of drug users and others in impoverished neighborhoods in different countries.

### "Big events" in these studied neighbourhoods and drug use

Some Big Events do unleash large-scale increases in drug or substance use, high-risk sex, and related HIV epidemics; and we do not know yet how to intervene during and after Big Events to prevent such outcomes. The need for research on Big Events as a top priority for HIV social and epidemiological research has been called for by our research team and by others [4,5].

### Poor territories, subsistence and social policies

When time in the workspace decreases (as it did for many subjects between 2001 and 2004), it leads to spending more time in the neighbourhood. Unemployment leads to people spending their time in the local areas in which they live. For them, material resources for subsistence are then obtained from local mediators who distribute money from public social security allotments [37-39]. After the crises, changes in the use of spaces where social life is carried out included a reduction of time spent in public spaces like neighbourhood streets, and an increase in time spent at home.

The crisis did not lead to increased drug use in these localities despite leading to reductions in work time and increases in time looking for work (at least for IDUs). It led to less time spent in neighbourhood streets and more time in housework. Those most impacted by decreased work time and increased time looking for work were most likely to increase time spent using drugs.

Importantly, our findings indicate that *in these neighbourhoods *of Greater Buenos Aires, *the Argentine crisis did not lead to an increase in drug users*, which somewhat contradicts our initial hypotheses. The number of IDUs and drug users stayed stable or decreased between 2001 and 2004. In fact, injecting may have decreased between 2001-2004.

Between 2001 and 2004, changes in time using drugs differed for non-IDUs and IDUs: the *mean hours *spent using drugs remained constant for non-IDUs, but declined among IDUs. Nevertheless, for IDUs, drug use continues to take up a lot of time even though injecting has diminished and is more hidden.

Risk of drug use and its social, physical and other harms (such as arrest, exposure to sexually transmitted infections, and the harms that drug use per se can inflict on some users) may have increased among those youth most affected by the crisis, particularly among non-IDUs. Although mean hours stayed stable, the time spent consuming drugs grew among those whose time at work decreased and among those whose job search time increased after the crisis. This suggests that drug use time increased for those who have difficulties entering or staying in the labour market.

Although the lack of increase in injection drug use is a hopeful result, this finding may be limited to these neighbourhoods. We propose a hypothesis based on these results, and recommend that research be conducted on this hypothesis in countries where Big Events take place: Traditionally less-impoverished and or less drug-impacted neighbourhoods may lack factors that protected Avellaneda. Two reasons lead us to suggest this hypothesis: First, Avellaneda neighbourhoods have benefited from outreach and other harm reduction programs conducted by Intercambios Civil Association since 1999. These programs reached many IDUs, non-IDUs and youth with prevention messages and supplies related to drug use and sexual practices [40-43]. Second, Avellaneda has been deeply impoverished and drug-impacted for decades, and thus may have already adapted to joblessness and poverty, and developed some collective cultural resiliency from years of coping with extreme poverty, spare time, "hustling" time, and economic decline.

Nevertheless, focusing on the country as a whole, there are signs of alienation and of a decrease in successful normative regulation of youth in studies conducted after the crises. These include increasing school dropout rates [44,45], and a rise in youth violence, particularly homicides in slum areas [46-48]. Such violence is particularly traumatic in Argentina since many families live with the effects of the dictatorship of 1976 - 1983. During the dictatorship, thousands of youth and adults were disappeared and, in many cases, tortured and/or killed. Their family members were often terrorized into silence. Furthermore, as Bastos et al [49] have noted, the traumas of the dictatorship period interact with a long-term high level of structural violence, inequity and disrespect for human dignity, which prevail in many Latin American countries. Thus, youth violence, and the lack of justice in many of these situations-particularly among the poorest victims- interacts with these pre-existing traumas to spread fear and alienation among additional youth, and this might lead some of them into substance use as a form of self-medication or escape. Research on how the effects of this fear and alienation differ for youth by neighbourhood (long-term-impoverished like Avellaneda, working class, middle class), and how time use, fear and alienation vary among youth with and without job and/or school time-commitments, should help us understand how to reduce drug-related harm in different circumstances.

## Limitations

This study was limited because it was organized only after the crises of 2001 - 2002. As a result, although retrospective time use data was validated by Juster et al [27] and by Stimson & Oppenheimer [50], recall error may have reduced the accuracy of reports about time use data "3 years ago." In addition, no ethnographic data are available for this earlier period.

Since this study was conducted under the pressure of time (in order to provide timely public health data if a disaster was brewing and also in order to minimize the length of the retrospective recall period), and also due to related limits on available research funding, the length and depth of the questionnaire were necessarily restricted. Data on alienation, hopelessness, and other psychosocial characteristics could thus not be obtained.

As field work occurred between 10 a.m. and 6 p.m. to assure better security conditions for the working team, the sampling may have under-recruited eligible subjects who were not available during these hours--which might include both people with stable jobs and drug users who sleep during the day. The small number of IDUs in the study limits what we can conclude about them. The fact that the sample is not a probability sample and the known limitations of self-report data also limit confidence in these findings. Another limitation is that the analyses only measured associations and therefore no causal relationship can be established. Nonetheless, the following conclusions regarding risk practices and the changes in time-use produced by the crisis seem consistent with what we observed in our ethnographic observations and focus groups.

## Conclusions

We conclude with three suggestions about future research: First, research about pathways--such as changed economic and social relationships and their associated implications for time use--through which crises and transitions can affect time use and drug use should be conducted. Second, time use methods should be more widely applied in studies of HIV risk and studies of drug use.

Finally, Big Events will continue to occur and, in some but not all cases, to precipitate large increases in drug use, drug injection, sex work, and related diseases [3,4,7]. We need to learn more about what determines such outcomes. We thus strongly urge that a program of social epidemiologic monitoring of risk behaviours, time use, norms, alienation, and related variables [3,4,7] be established, and data collected, in potential flash-points prior to potential Big Events, and that resources be allocated in advance for follow-up studies during and after such crises. If this is done, we will be able to conduct well-planned studies in timely fashion and develop the knowledge we need to prevent future Big Events from leading to epidemic outbreaks.

## List of Abbreviations

IDU: Injection Drug User;

## Competing interests

The authors declare that they have no competing interests.

## Authors' contributions

DR helped design the study, drafted the manuscript, participated in data collection and analysis. DZS wrote an initial draft of the manuscript, conducted the ethnography, and participated in bibliographic search. MPP participated in data collection and analysis. GT participated in the design of the study. MB performed some statistical analysis. PMG provided essential advice on study design and ethnography and assisted in writing the questionnaire. MS provided guidance on field methods for conducting the survey and assisted in writing the questionnaire. SRF conceived the study, and participated in its design and coordination and helped to draft the manuscript. All authors took part in reading and revising the final manuscript.
